# Green Approaches for Preparation of Natural Deep Eutectic Solvents for Determination of As, Cd, and Pb in Plant Samples by ICP-MS

**DOI:** 10.1021/acsomega.5c03345

**Published:** 2025-06-11

**Authors:** Sabrina S. Ferreira, Rafaela S. Lamarca, Leandro S. Silva, Thiago A. L. Burgo, Paulo C. F. Lima Gomes, Clarice D. B. Amaral, Jose O. Fernandes, Sara C. Cunha, Mario H. Gonzalez

**Affiliations:** † Department of Chemistry and Environmental Science, 28108 São Paulo State University (UNESP), São José do Rio Preto, São Paulo 15054-000, Brazil; ‡ Department of Analytical Chemistry, Physical Chemistry and Inorganic Chemistry, Institute of Chemistry, São Paulo State University (UNESP), Araraquara, São Paulo 14800-060, Brazil; § Department of Chemistry, 28122 Federal University of Paraná (UFPR), Curitiba, Paraná 81531-980, Brazil; ∥ LAQV-REQUIMTE, Laboratory of Bromatology and Hydrology, Faculty of Pharmacy, University of Porto, Porto 4050-313, Portugal

## Abstract

Natural deep eutectic solvents (NADESs) and amino acid-based deep eutectic solvents (AADESs) are considered green solvents due to their biodegradable characteristics, low toxicity, low cost, simple preparation and handling, negligible volatility, and, especially, modulable physicochemical properties (density, viscosity, and polarity). In this work, two green methods for preparation of NADES and AADES were developed and evaluated with characterization of the solvents produced. Eutectic solvents based on citric acid/β-alanine/water (Ala-CA), citric acid/xylitol/water (Xyl-CA), and citric acid/malic acid/water (MA-CA) were prepared either by stirring without heating or by using a rotary evaporator under reduced pressure. Comparison was made to the solvent obtained by the reference preparation method involving stirring with heating. Fourier transform infrared spectroscopy (FTIR), in attenuated total reflectance (ATR) mode, showed wavenumber shifts, such as that of the C=O band from 1696 to 1710 cm^–1^, indicating the occurrence of interactions for formation of the solvents. Determination of the physicochemical properties of the solvents revealed significant differences between the methods, with viscosity values from 5.99 ± 0.02 to 10.9 ± 0.01 mPa·s for the stirring method and from 18.9 ± 0.03 to 26.02 ± 0.02 mPa·s for the rotary evaporator method. The melting points obtained by differential scanning calorimetry (DSC) were 240–251 K (stirring method) and 223 K (rotary evaporator method). The deep eutectic natures of the solvents were confirmed by using phase diagrams and thermodynamic calculations to estimate the ideal eutectic points of the ternary mixtures. These solvents provided satisfactory results in microwave-assisted extraction of plant material, with recoveries of 87–106% for determination of Cd and Pb, using the NADES prepared by the stirring method, and for As and Pb, using the AADES prepared by the stirring and rotary evaporator methods. The results demonstrated the effectiveness of the proposed methods for the preparation of NADES/AADES, with the potential to modulate the physical properties, such as viscosity and melting point, of these water-based solvents. Evaluation using EcoScale resulted in 99 points for the stirring method and 98 points for the rotary evaporator method, representing high green scores for these methods.

## Graphical abstract

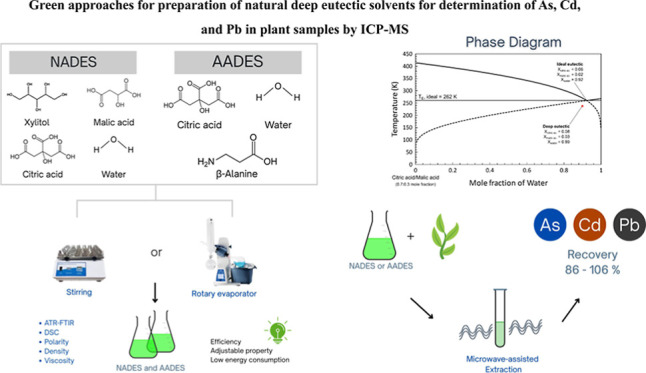

## 1 Introduction

Actions aimed at reducing the production, use, and exposure potential of harmful chemicals are essential for achieving sustainability goals. Unfortunately, conventional analytical methods often worsen environmental issues, because the production and use of toxic solvents contribute to the generation of hazardous waste during the analytical process.^1–3^ For this reason, significant efforts are being made to develop new sustainable solvents to replace toxic ones, aiming at reducing the environmental impacts of analytical methodologies while maintaining the quality of analytical results.

A major challenge in green analytical chemistry (GAC) is the design and synthesis of sustainable solvents suitable for use with a wide variety of solutes. A sustainable solvent should comply with the principles of GAC, considering aspects including waste disposal, impacts on the environment and human health, and safety of use.^4^

Deep eutectic solvents (DESs) are currently considered a sustainable and economically attractive option, due to their low toxicity, biodegradability, and affordable synthesis.^5,6^ A DES is a mixture of two or more organic compounds at a ratio close to the eutectic point, with a melting point lower than for each of the precursors alone.^7^ These solvents are known as natural deep eutectic solvents (NADESs), when natural compounds such as sugars, organic acids, amines, and amino acids are used in the synthesis.^8^ A NADES can be more strictly classified as an amino acid-based deep eutectic solvent (AADES), when an amino acid is used as one of the solvent precursors.^9,10^

The components of DES such as NADES and AADES interact primarily by means of hydrogen bonds, with one of the precursors serving as a hydrogen-bond donor (HBD) and the other as a hydrogen-bond acceptor (HBA). The intermolecular hydrogen bonding network allows for charge delocalization, which is described as the cause of the decreased melting points of DES.^8,11^ Changing the molar ratio of the precursors, or adding water to the solvent, modifies the interactions between the components, resulting in changes in physicochemical properties such as density, viscosity, and melting temperature.^8,12^

Since the discovery of DES and NADES, many studies have explored different combinations of precursors, different syntheses, and optimization of parameters such as the component ratio and water volume.^13^ The main methods described in the literature for the preparation of DES are stirring and heating, lyophilization, vacuum evaporation, microwave irradiation, and ultrasonication. The stirring and heating method involves mixing two separate components in the presence of water, followed by stirring and heating the solution on a magnetic stirrer, until a clear liquid is formed.^14^ In the evaporation method, the components are solubilized in water and evaporated at a temperature of approximately 50 °C, using a rotary evaporator, followed by storage of the resulting liquid in a desiccator until it reaches constant weight.^8^ In microwave-assisted NADES preparation, the precursors are irradiated in a closed system, at controlled power and temperature.^15,16^ Solvent formation can also be induced by sound waves in ultrasound-assisted NADES preparation.^16^

Due to their adaptability, DES and NADES are gaining popularity in analytical chemistry, as well as in areas such as catalysis, electrochemistry, materials chemistry, and organic synthesis.^14,17^ In the last ten years, there has been a significant amount of research on the use of DES and NADES as extraction solvents employed in the preparation of solid and liquid samples.^13,18^ Most of the studies have focused on the extraction of organic substances such as pesticides, drugs, bioactive substances, and phenolics.^19,20^ The use of DES and NADES for the extraction of inorganic analytes has also been investigated in several studies.^21–23^ Liquid-phase microextraction (LPME), ultrasound-assisted microextraction (UAME), and microwave-assisted extraction (MAE) are frequently mentioned in the literature, among many other sample preparation techniques using DES as extraction solvents.^10,24,25^

The number of studies exploring the applicability of DES has been increasing annually, but the influence of the physicochemical properties of DES has not yet been fully elucidated.^26^ Therefore, the aim of this study was to investigate the physicochemical properties of NADES and AADES based on xylitol, β-alanine, and citric acid, prepared using two different routes. Investigation was made of the physicochemical properties of these solvents, including freezing point, density, viscosity, and polarity, and the solvents were compared with others prepared using the widely employed stirring with a heating method. The sustainability and green characteristics of the developed preparation methods of the solvents were evaluated using the EcoScale metric.^27^ In addition, the extractive capacities of the solvents prepared by the proposed methods were evaluated in the analysis of plant tissue material using microwave-assisted extraction (MAE), followed by determination of toxic elements by ICP-MS.

## 2 Material and Methods

### 2.1 Reagents

The NADES and AADES were synthesized using xylitol (99% purity, CAS 87-99-0), citric acid (99.5% purity, CAS 77-92-9), dl-malic acid (99% purity, CAS 6915-15-7), and β-alanine (99% purity, CAS number 107-95-9). All of these chemicals were purchased from Sigma-Aldrich (MO, USA). Ultrapure deionized water (18 MΩ cm) was obtained from a Milli-Q system (ICW-3000, Merck KGaA, Darmstadt, Hessen, Germany).

### 2.2 Stirring Preparation Method

The stirring method for preparation of NADES and AADES based on xylitol, β-alanine, citric acid, and malic acid was performed considering the method and optimal proportions proposed by Santana et al. and Guimarães et al.^21,28^ The components of each solvent were weighed according to the molar ratios and compositions shown in Table 1, followed by mixing. Each mixture was then kept under stirring on a shaker table (model SL-180/DT, Solab) at 220 rpm for 2 h. After preparation, the NADESs were stored in desiccators, prior to subsequent characterization. Evaluation was also made of preparation using a stirring period of 20 min.

**Table 1:** Composition Information for the Prepared NADES and AADES

solvent	mass ratio (% w w^–1^)	molar ratio	composition
Xyl-CA NADES	42:13:45	3:1:30	citric acid:xylitol:water
MA-CA NADES	42:13:45	2:1:26	citric acid:malic acid:water
Ala-CA AADES	43.75:12.5:43.75	2:1:17	citric acid:β-alanine:water

### 2.3 Preparation under Reduced Pressure in a Rotary Evaporator

The components of NADES and AADES were weighed out according to the molar ratios shown in Table 1, followed by mixing. The mixtures were kept for 25 min in a rotary evaporator (model RD-180, Gehaka, São Paulo, Brazil) operated at 110 rpm, 50 °C, and −760 mm Hg. After preparation, the materials were transferred to conic tubes and stored in desiccators, prior to subsequent characterization.

### 2.4 Preparation by Stirring with Heating

The components of the NADES and AADES were weighed out according to the molar ratios shown in Table 1, followed by mixing and heating in a water bath for 2 h, at 50 °C, with magnetic stirring at 220 rpm (AccuPlate Hot plate Stirrer, Labnet, Edison, USA), as described by Santana et al. and Guimarães et al.^21,28^ The prepared materials were stored in desiccators prior to subsequent characterization. This is the most widely used method for preparing DES and will be discussed as the standard procedure for obtaining these solvents.

### 2.5 Characterization of the NADES and AADES

Infrared spectra of the NADES and AADES were obtained using a Fourier transform infrared spectrometer (Spectrum Two, PerkinElmer, USA) operated in attenuated total reflectance (ATR) mode, in the range 4000 to 400 cm^–1^, with a resolution of 4 cm^–1^ and an accumulation of 64 scans.

Density measurements of the NADES and AADES were performed (in triplicate) using a pycnometer calibrated with water, at 24 °C, and an analytical balance with a precision of ±0.0001 g (model AG200, Gehaka, São Paulo, Brazil). Viscosity measurements of the solvents were made (in triplicate) using a Cannon-Fenske viscometer calibrated with water, at 24 °C.

The melting points of the solvents were determined by differential scanning calorimetry (DSC), using a TA2010 instrument controlled by a TA5000 module (TA Instruments, USA). A 10 mg portion of the sample was placed on an aluminum support and heated from −100 to 400 °C, at 10 °C min^–1^, under a flow of N_2_ (100 mL min^–1^).

The polarities of the solvents were determined by solvatochromic analysis using Reichardt’s dye. Solvent-dye solutions were prepared (in triplicate) by diluting 10 μL of the dye solution (10 mM Reichardt’s dye prepared in methanol) in 1.0 mL of NADES or AADES, followed by ultrasonication during 20 min to ensure complete dissolution of the dye. The solvent-dye solutions were diluted and analyzed using a scanning UV–vis spectrophotometer (UV-2600, Shimadzu, Japan) operated in the range of 200–600 nm. The Dimroth–Reichardt parameter was calculated based on the molar energy value, *E*_t_ (30), in kcal mol^–1^, according to eq 1,^29^ where λ_max_ is the maximum absorption wavelength.

1 $$E_{t}\;\left(30\right)=\frac{28591}{\lambda_{\max}}$$

### 2.6 Sample Preparation and Analysis by ICP-MS

#### 2.6.1 Microwave-Assisted Extraction (MAE) with NADES and AADES

The solvents prepared by stirring and rotary evaporator were used in a microwave-assisted extraction method to prepare a sample of a forage grass reference material (Brachiaria brizantha cv. Marandu, E1001a) supplied by the Brazilian Agricultural Research Corporation (Embrapa, São Paulo, Brazil), following the procedures described by Santana et al. and Guimarães et al.^21,28^ For the Xyl-CA and MA-CA NADES, 90 mg of the sample and 9 mL of the solvent were used, with the following heating program: (I) 2 min ramp to 100 °C and (II) maintaining at 100 °C for 18 min (Santana, 2020). For the Ala-CA AADES, 200 mg of the sample and 5 mL of the solvent were used, with the heating program: (I) 2 min ramp to 100 °C and (II) maintaining at 100 °C for 40 min (Guimarães et al., 2022). After extraction, the suspensions were filtered, and the supernatants were appropriately diluted in ultrapure deionized water, prior to analysis by ICP-MS. The procedure was performed in triplicate.

#### 2.6.2 ICP-MS Analysis

The extracts were analyzed by using a NexION 300X ICP-MS system (PerkinElmer, USA) equipped with a concentric nebulizer, a cyclonic nebulization chamber, and a quartz torch with a quartz injector tube (2.0 mm). The instrumental parameters were adjusted according to the manufacturer’s recommendations. The isotopes ^75^As^+^, ^111^Cd^+^, and ^208^Pb^+^ were analyzed in standard mode. The ICP-MS operating conditions are listed in Table 2. Calibration curves were prepared using dilutions of a 1000 mg L^–1^ stock standard solution of all of the analytes (Sigma-Aldrich, USA). For matrix matching, the calibration curves for analysis of the extracts were prepared using the standards in a NADES medium (1% v v^–1^).

**Table 2:** Instrumental Parameters for the Analysis of ^75^As^+^, ^111^Cd^+^, and ^208^Pb^+^ by ICP-MS

instrumental parameters	
radiofrequency power	1600 W
plasma gas flow	18 L min^–1^
auxiliary gas flow	1.2 L min^–1^
nebulizer gas flow	1.0 mL min^–1^
sample uptake rate	0.70 mL min^–1^

method parameters	
sweeps/reading	50
readings/replicate	1
replicates	3
dwell time	25 s
RPq^a^	0.25
analytical calibration range	0.50–20 μg L^–1^

a RPq: quadrupole dynamic bandpass tuning parameter.

## 3 Results and Discussion

In this study, two preparation methods for NADES and AADES were developed: stirring and a rotary evaporator. These methods were compared with preparation by stirring and heating, which is a method commonly reported in the literature.

### 3.1 Eutectic Characteristics and Physicochemical Properties

FTIR analysis was employed to investigate the intermolecular interactions and functional groups of the eutectic mixtures. The FTIR spectra of the precursors and NADES and AADES prepared by the three different methods (stirring, stirring with heating, and under reduced pressure in a rotary evaporator) are presented in Figure 1.

**Figure 1:**
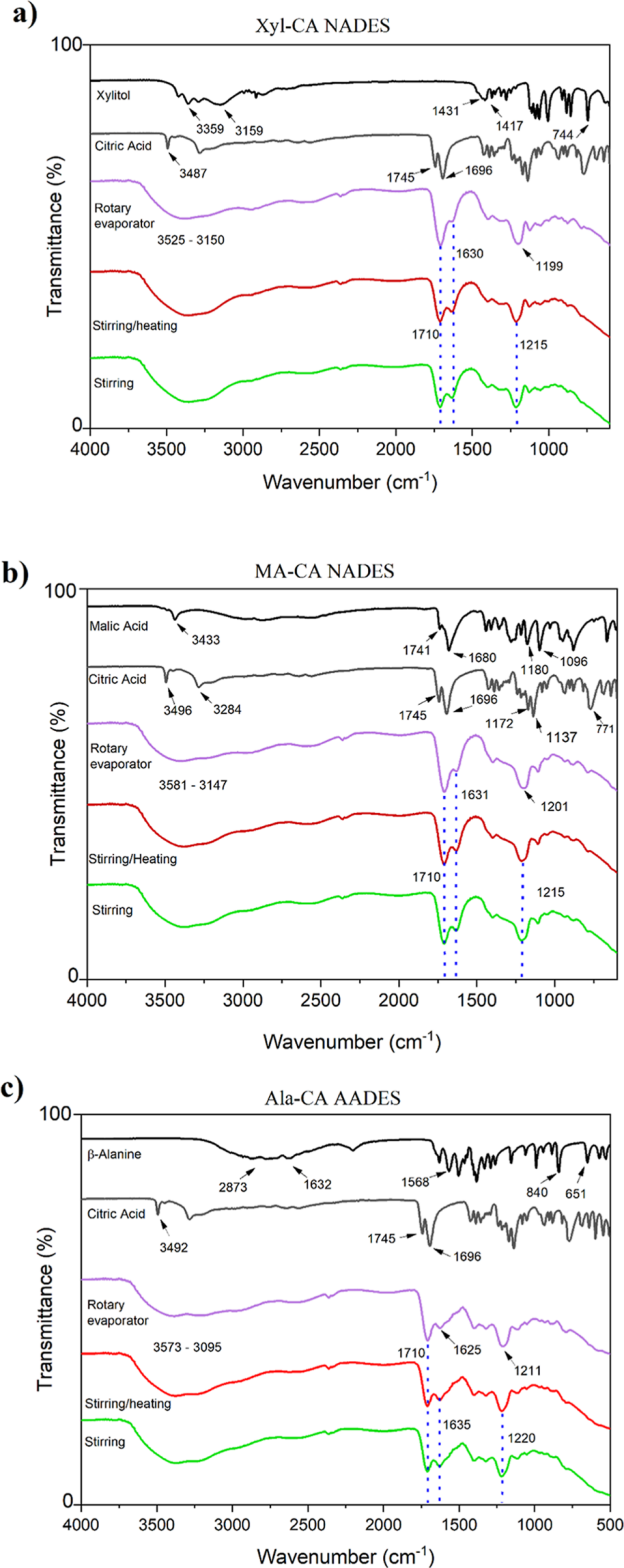
ATR-FTIR spectra of the initial reagents and the NADES and AADES prepared using the different methods for (a) Xyl-CA, (b) MA-CA, and (c) Ala-CA.

The infrared spectra of the Xyl-CA NADES and the individual components (Figure 1(a)) showed bands characteristic of the functional groups of each precursor. Citric acid presented an absorption band at 3487 cm^–1^, corresponding to stretching of the free –OH groups of the molecule, together with bands at 1745 and 1696 cm^–1^, attributed to the stretching of the C–O–H and C=O bonds, respectively. For xylitol, absorption bands at 3426, 3367, and 3229 cm^–1^ corresponded to free –OH groups in the molecule, while peaks at 1431 and 743 cm^–1^ were assigned to in-plane and out-of-plane flexions of –OH, respectively. The spectra for the Xyl-CA NADES obtained using the three different preparation methods showed an intense absorption band between 3525 and 3150 cm^–1^, corresponding to the stretching of –OH groups, together with a band at 1630 cm^–1^, attributed to an angular deformation of –OH groups, which could be explained by the presence of water in the composition.

The evidence for intermolecular interactions in the formation of eutectic solvents has been mainly based on shifts of peaks characteristic of the C=O and the O–H bonds. The formation of the evaluated solvents was evidenced by a shift of the C=O band from 1696 to 1710 cm^–1^,^21,28,30^ suggesting an increase in the electron density of carbonyl oxygen by the formation of hydrogen bonds between the precursors.

Figure 1(b) shows the spectrum for the malic acid precursor of MA-CA NADES, highlighting a band at 3471 cm^–1^, corresponding to the –OH groups of the molecule, a C=O stretching band at 1680 cm^–1^, and a C–H_2_ stretching band at 1180 cm^–1^. The same features observed in the spectrum for the Xyl-CA NADES were found for the MA-CA NADES, with an intense absorption band between 3581 and 3147 cm^–1^ corresponding to the –OH stretching, a band at 1631 cm^–1^ attributed to the angular deformation of –OH, and a shift of the C=O band from 1696 to 1710 cm^–1^.

Figure 1(c) shows the spectra of Ala-CA AADES and its precursors. Characteristic bands of β-alanine at around 2873 and 2632 cm^–1^ corresponded to the amino acid hydrogen bonds, while peaks at 1568, 846, and 651 cm^–1^ could be attributed to the carbonyl (C=O), amine (N–H), and carboxyl (–COO) groups, respectively. As observed for the two NADES mentioned above, the Ala-CA AADES spectrum presented bands corresponding to –OH groups at around 3550 and 3000 cm^–1^ ^31^ and a C=O peak upshift from 1696 to 1710 cm^–1^.

Analysis of the spectra for the products from the different preparation methods revealed the formation of solvents when using the stirring method and the rotary evaporator under the reduced pressure method. The spectra differed only in the region where an absorption band at 1630 cm^–1^ was observed, corresponding to the angular deformation of –OH groups. The spectrum for the solvent obtained by the rotary evaporator under the reduced pressure method showed lower intensity of the band in this region, which could be explained by the greater loss of water for this method, which was reflected in the higher viscosity of the solvent (see Table 3). Overall, the FTIR spectra of the solvents prepared by the two proposed methods presented the same profile as solvents of the same composition prepared by the stirring with heating method, reported by Santana et al. and Guimarães et al.^21,28^

**Table 3:** Melting Points of the Solvents Prepared by the Three Methods

melting point (K)			
solvent	stirring	stirring/heating	rotary evaporator
Xyl-CA NADES	245	242	
MA-CA NADES	240	238	
Ala-CA AADES	251	251	223

The limit between the formation of the eutectic mixture and only solubilization of the components was investigated by applying the stirring procedure for a time until complete homogenization of the precursors, which was 20 min. The FTIR spectrum of the mixture obtained (Figure 2) showed no shift of the C=O bond peak at 1696 cm^–1^ to longer wavelengths, in addition to the peak having a lower intensity. This suggested that there was no significant increase in the electron density of carbonyl oxygen for the formation of hydrogen bonds between the precursors, which was a characteristic expected for effective formation of the solvent, under the conditions employed.

**Figure 2:**
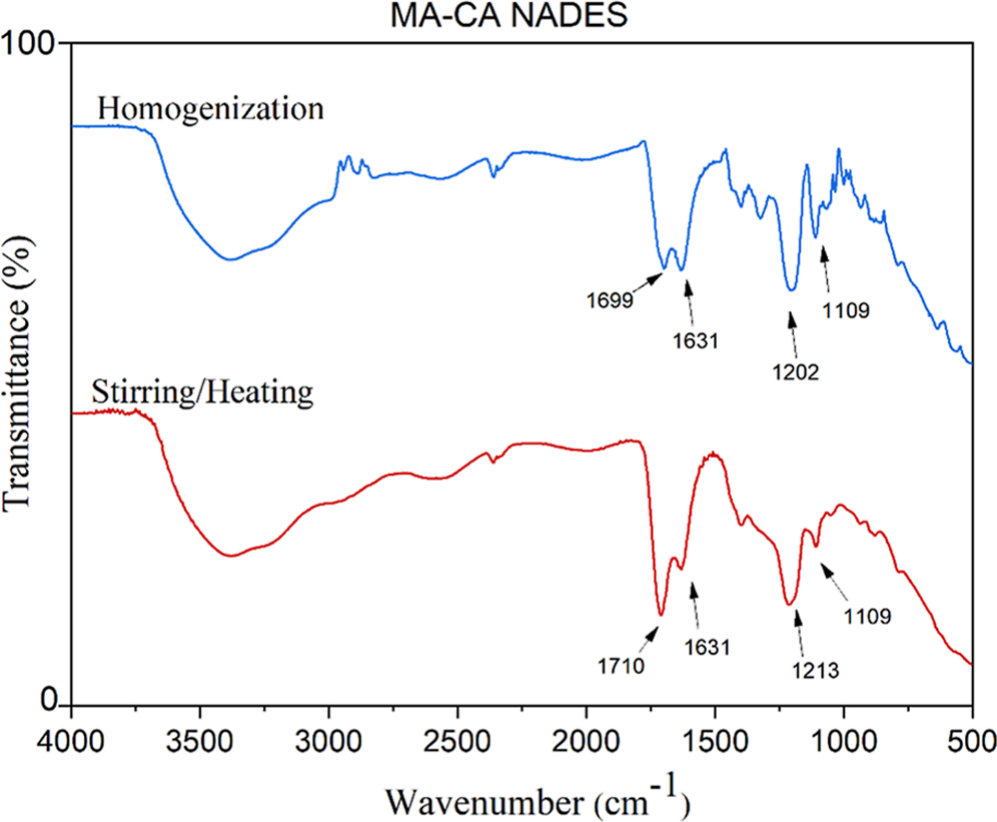
Comparison of the ATR-FTIR spectrum for the MA-CA NADES prepared by the stirring and heating method and the spectrum for the same components only solubilized under stirring.

It has been suggested that the formation of a deep eutectic solvent cannot be confirmed only by evidence of hydrogen-bond formation and by the eutectic point of the mixture being lower than the melting points of the individual components. It is necessary for the solvent to present a eutectic point temperature below the eutectic temperature of the ideal liquid mixture, with significant negative deviations from ideality, for it to be considered a deep eutectic solvent.^26^ Therefore, analyses were performed to identify the melting points, calculate the ideal eutectic points, and obtain the phase diagrams of the proposed mixtures.

The NADES and AADES were submitted to DSC measurements at low temperature to identify the melting points of the mixtures obtained by the different preparation methods. Figure 3 shows the DSC curves for the Ala-CA AADES. The Xyl-CA and MA-CA NADES prepared by using a rotary evaporator under reduced pressure showed instability in solution, with phase separation occurring after 15 days of storage. This observed phase separation suggested disruption of the intermolecular interactions of the solvent, which precluded accurate melting point determination for these systems. The melting points obtained for NADES and AADES prepared using the three different methods are presented in Table 3.

**Figure 3:**
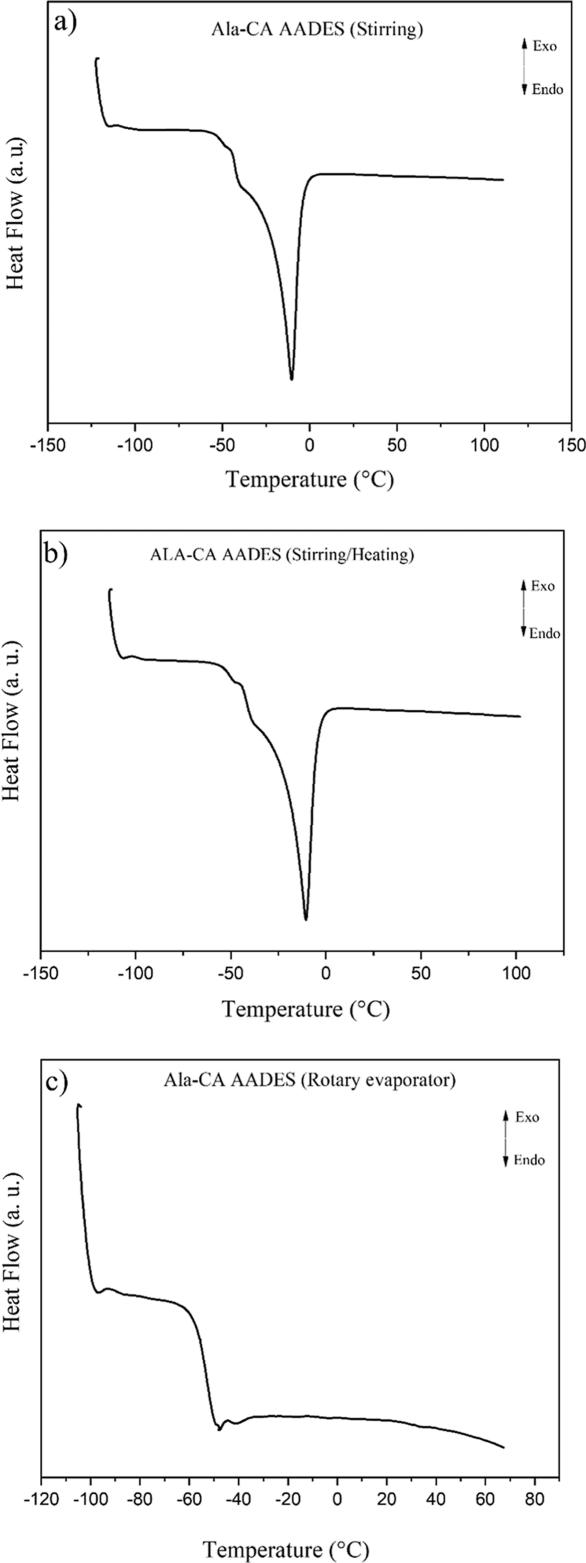
DSC curves for the Ala-CA AADES obtained using the different methods: (a) stirring, (b) stirring/heating, and (c) rotary evaporator under reduced pressure.

Experimental determination of phase diagrams for binary and/or ternary systems is usually time-consuming, costly, and labor-intensive.^32^ On the other hand, computer calculations can often assist in estimating the eutectic points for ideal solutions. In fact, since the ideal solution eutectic point can be estimated from classical thermodynamics, it is used as an initial indicator for the preparation of deep eutectic solvents.^26^ The chemical potential of a component A in an ideal solution at equilibrium is given as follows:

2 $$\mu_{A}=\mu_{A}^{*}+RT\ln X_{A}$$

where μ_A_^*^ is the chemical potential of pure A, *R* is the gas constant, *T* is the temperature, and *X*_A_ is the mole fraction of A. A solid solute B in contact with A will dissolve until saturation, which is also in a state of equilibrium. Therefore, the ideal solubility of B can be estimated considering undissolved *B* (μ_B_^*^(s)) in equilibrium with dissolved B in solution (μ_*B*_(l)):

3 $$\mu_{B}^{*}\left(s\right)=\mu_{B}^{*}\left(l\right)+RT\ln X_{B}$$

Equation 4, derived from the chemical potential, is the expression for the ideal solubility, which can also be derived using the Gibbs energy change in a thermodynamic cycle^33,34^ (see resolution in the Supporting Information).

4 $$\ln X_{B}=\frac{-\Delta_{fus}H_{B}\left(T^{*}\right)}{R}\left[\frac{1}{T}-\frac{1}{T^{*}}\right]+\frac{\Delta_{fus}C_{p\left(B\right)}}{R}\left[\ln\frac{T}{T^{*}}+\frac{T^{*}}{T}-1\right]$$

For real solutions, the mole fraction of B is simply replaced by its activity (*a*_B_ = *X*_B_ × γ_B_, where γ_B_ is the activity coefficient of B). Equation 4 can be used to construct the *liquidus* curves^35,36^ of temperature–composition phase diagrams of ideal solutions. In an ideal solution, the enthalpy of mixing is zero because the interactions between the various species in the mixture are effectively the same.

For most compounds, the difference between the molar heat capacity of compound *B* in the liquid and solid phases is negligible, when compared to Δ_fus_ *H*_B_. The following equation is a reasonable approximation for estimating the *liquidus* curves of ideal solutions

5 $$\ln X_{B}=\frac{-\Delta_{fus}H_{B}\left(T^{*}\right)}{R}\left[\frac{1}{T}-\frac{1}{T^{*}}\right]$$

Equation 5 was used to construct the *liquidus* plots for binary systems composed of citric acid/water, citric acid/malic acid, and malic acid/water. The thermodynamic properties of citric acid monohydrate were chosen for the citric acid/water phase diagram, while the properties of pure citric acid were used for the citric acid/malic acid plot.

Equation 5 was used to construct the temperature–composition phase diagram of an ideal solution composed of citric acid, malic acid, and water (Figure 4). Instead of plotting a 3D space model, a 2D plot design was used for this ternary system, since this could also be conveniently performed using experimental data.^38^ The *liquidus* plot was constructed as a function of the mole fraction of water added but starting from an ideal mixture of the two acids with fixed composition. This initial mole fraction (isopleth) was chosen based on preliminary experimental results, while the melting temperature for the citric acid/malic acid binary system was extracted from the citric acid/malic acid binary phase diagram.

**Figure 4:**
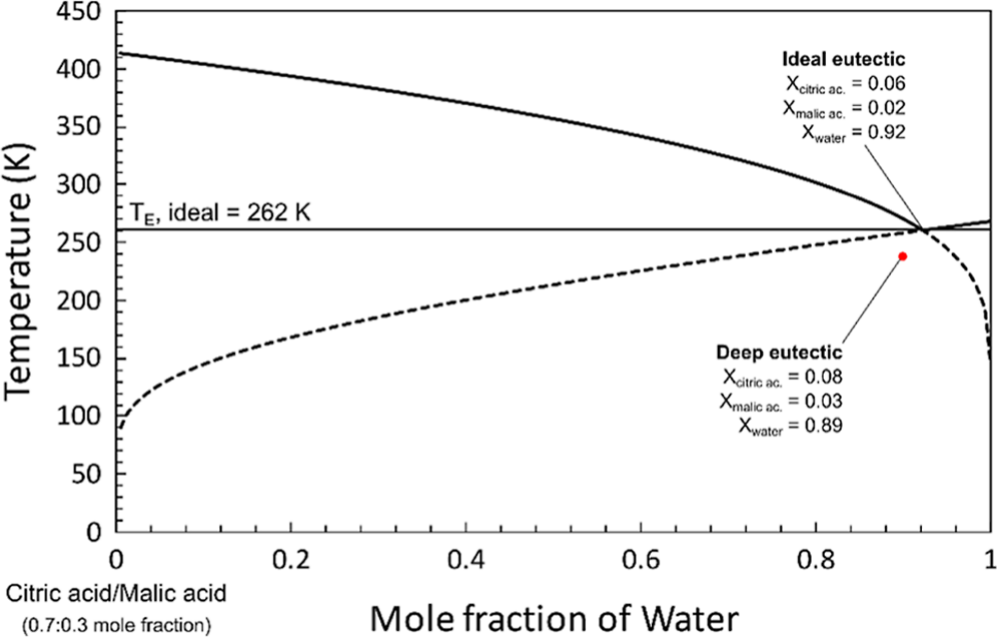
*Liquidus* curve for an ideal solution of 0.7:0.3 (mole fraction) citric acid/malic acid, as a function of water content.

Despite the expected deviations from ideality, the eutectic point for the citric acid/water diagram treated as an ideal solution (262 K) is very close to that found experimentally (265 K).^37^ For the xylitol- and malic acid-based NADESs, the same ideal eutectic point of 262 K was obtained, while for the β-alanine-based AADES, the ideal eutectic point was 260 K.

Density, viscosity, and polarity are important properties to be determined, as they define the behavior of solvents and guide the selection of suitable applications.^39^ The density and viscosity values obtained for NADES and AADES prepared by the different methods are presented in Table 4.

**Table 4:** Densities and Viscosities of the NADES and AADES Prepared at a Temperature of 24 °C (Mean ± Standard Deviation, *n* = 3)

preparation method	density (g mL^–1^)	viscosity (mPa s)
Xyl-CA NADES		
stirring	1.25 ± 0.01	7.46 ± 0.02
stirring/heating	1.26 ± 0.01	6.90 ± 0.01
rotary evaporator	1.30 ± 0.01	21.5 ± 0.05
MA-CA NADES		
stirring	1.26 ± 0.01	5.99 ± 0.02
stirring/heating	1.27 ± 0.01	5.83 ± 0.05
rotary evaporator	1.34 ± 0.01	18.9 ± 0.3
Ala-CA AADES		
stirring	1.26 ± 0.01	10.9 ± 0.01
stirring/heating	1.27 ± 0.01	11.2 ± 0.08
rotary evaporator	1.30 ± 0.01	26.2 ± 0.2

The density values indicated that there was no significant difference (Student’s *t*-test for independent samples, 95% confidence level, *p* > 0.05) between the stirring and stirring/heating methods or between the stirring/heating and rotary evaporator under reduced pressure methods. However, there was a statistically significant difference (*p* < 0.05) between the stirring and rotary evaporator under reduced pressure methods, although in practice the difference would have little effect on the solvent properties. The viscosity values of the solvents increased in the order stirring/heating < stirring < rotary evaporator under reduced pressure, with a statistically significant difference (Student’s *t*-test for independent samples, 95% confidence level, *p* < 0.05) for the rotary evaporator under the reduced pressure method. This could be explained by the differences in the percentages of water evaporated in the methods. Hence, the greater water loss from the mixture in the rotary evaporator under the reduced pressure method could explain the higher viscosity and density values.

For application in the preparation of samples for inorganic elemental analysis by spectroanalytical techniques, solvents with low viscosities are desirable because the phenomenon of mass transfer between the sample and the extractor medium is facilitated. In addition, considering techniques that employ plasma sources, such as ICP-MS and ICP-OES, less viscous solvents favor transport of the sample to the plasma.^40^ Viscosity is a property that varies as a function of the temperature. Increase of the temperature implies greater molecular activity and mobility, since the internal resistance of the molecules decreases and the molecules flow more easily, usually resulting in decreased viscosity.^41^ Savi et al.^42^ studied viscosity behavior, as a function of temperature, for different NADESs based on lactic acid and malic acid. For all the NADESs, an inverse relation between the temperature and viscosity was observed in the temperature range from 293.15 to 344.15 K. Hence, extraction methods that require heating the system can assist the application of slightly more viscous solvents, when a low water content is of interest for the extraction of more nonpolar analytes.

Knowing the polarity of NADES and AADES is important for understanding their behavior and predicting their solvation and extraction capacities in different matrices. The polarity of these solvents can be characterized using solvatochromic probes that enable observation of the changes in the UV–vis spectroscopic responses resulting from the interaction between the probe molecule and the molecules that compose the solvent.^43,44^ Reichardt’s dye is an example of a negative solvatochromic probe, where a shift of the absorption band to shorter wavelengths indicates increased solvent polarity.

Solute–solvent interactions are commonly measured using the Dimroth and Reichardt *E*_t_ (30) scale, which represents the intermolecular charge transfer of Reichardt’s dye and its absorption band. Higher *E*_t_ (30) values indicate higher polarity of the solvent.^43,44^ The *E*_t_ (30) values obtained for the different compositions of precursors and the three methods of preparation are listed in Table 5.

**Table 5:** *E*_t_ (30) and λ_max_ Values for the NADES and AADES Prepared by the Different Methods

	stirring		stirring/heating		rotary evaporator	
solvent	λ (nm)	*E*_t_ (30) (kcal mol^–1^)	λ (nm)	*E*_t_ (30) (kcal mol^–1^)	λ (nm)	*E*_t_ (30) (kcal mol^–1^)
Xyl-CA NADES	306	93.6	305	93.7	303	94.4
MA-CA NADES	303	94.5	302	94.7	300	95.5
Ala-CA AADES	304	94.2	304	94.0	304	94.2

The *E*_t_ (30) values were in the range from 93.6 to 95.5 kcal mol^–1^, with λ_max_ between 300 and 306 nm, indicating solvents with high polarity, since the *E*_t_ (30) value for pure water is 63.1 kcal mol^–1^.^44^ These results were in accordance with the values of 304 ± 0.7 nm and 93.9 ± 0.22 kcal mol^–1^ found by Guimarães et al.^28^ for AADES based on citric acid, alanine, and water, using the same proportions and method of preparation employed in this study. Therefore, it could be inferred that regardless of the method of preparation, the polarity remains the same; therefore, this is a physicochemical parameter that is independent of the route of preparation. Guimarães et al.^28^ evaluated the polarity of citric acid-based AADES prepared with different precursors, showing that the polarity changed with the use of different HBDs. In this study, the presence of citric acid as a component in a greater proportion in the three solvents evaluated could provide an explanation for their similar polar characteristics.

### 3.2 Application in the Elemental Analysis of Plant Material

The solvents prepared using the different procedures were employed in the MAE method applied to the extraction of a sample of a forage grass reference material (B. brizantha cv. Marandu, E1001a), to evaluate the differences between the preparation methods in terms of effective extraction. The limits of detection (LOD) and quantification (LOQ) were calculated considering measurements of the blank solution (*S*_blank_) and the slope of the calibration curve (*a*), according to the equations: LOD = 3 (*S*_blank_)/*a* and LOQ = 10 (*S*_blank_)/*a*. The LOD and LOQ values are shown in Table 6.

**Table 6:** Comparison of the LOD and LOQ Values Obtained for Determination of As, Cd, and Pb by ICP-MS, after Extraction Using Microwave-Assisted Acid Digestion (MW-AD) and Microwave-Assisted Extraction (MAE) with NADES and AADES Prepared by the Stirring, Stirring/Heating, and Rotary Evaporator Methods^a^

			As		Cd		Pb		
solvent preparation method	solvent	sample preparation method	LOD (mg kg^–1^)	LOQ (mg kg^–1^)	LOD (mg kg^–1^)	LOQ (mg kg^–1^)	LOD (mg kg^–1^)	LOQ (mg kg^–1^)	reference
	HNO_3_	MW-AD	0.0001	0.0006	0.0009	0.003	0.08	0.2	Brito et al.^45^
stirring/heating	Xyl-CA	MAE	0.008*	0.03*	0.05*	0.2*	1.3*	4.3*	Santana et al. and Guimarães et al.^21,28^
	MA-CA		0.02*	0.07*	0.002*	0.006*	2.6*	7.1*	
	Ala-CA		0.02	0.05					
stirring	Xyl-CA	MAE	0.01	0.04	0.01	0.02	0.01	0.4	
	MA-CA		0.06	0.2	0.01	0.02	0.08	0.2	this study
	Ala-CA		0.02	0.06	0.02	0.05	0.01	0.04	
rotary evaporator	Xyl-CA	MAE	0.1	0.4	0.02	0.05	0.01	0.04	
	MA-CA		0.08	0.2	0.01	0.02	0.04	0.1	this study
	Ala-CA		0.01	0.03	0.01	0.02	0.07	0.02	

a Data: * values in μg kg^–1^.

The LOD values obtained for As using NADES and AADES prepared by stirring were lower than those using the solvents prepared by the rotary evaporator method. For Cd, the LOD values showed no difference between the solvents prepared by stirring or rotary evaporator methods. For Pb, the values obtained using the solvents prepared with the rotary evaporator were lower than those using the solvents prepared with stirring. In general, the LOD values obtained using the NADES and AADES prepared by the stirring and rotary evaporator methods were higher than those obtained using solvents prepared by stirring/heating or nitric acid.^21,45^ The relative standard deviation (RSD %) obtained for the analyses using the solvents prepared by stirring ranged from 1 to 9%, while for most of the solvents prepared by the rotary evaporator, the variation was from 1 to 8%. An exception was the MA-CA NADES prepared by a rotary evaporator, where the range was from 1 to 16%. The recoveries obtained using the Xyl-CA and MA-CA NADES and the Ala-CA AADES are presented in Figure 5. Recoveries in the range of 80 to 110% were considered acceptable.

**Figure 5:**
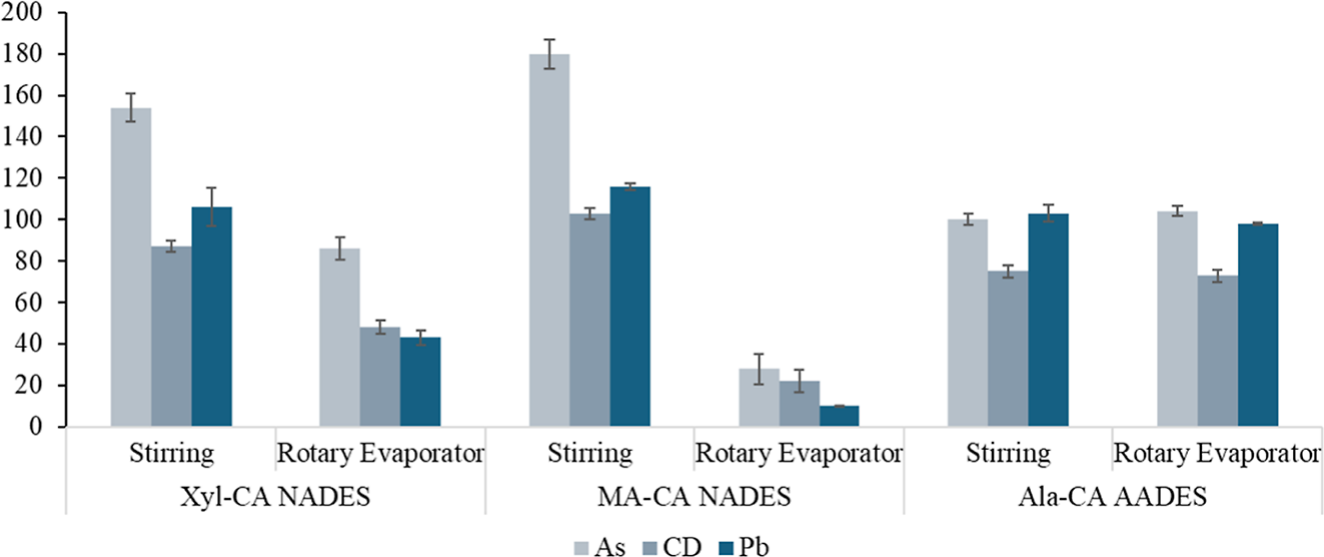
Recovery values (%) for As, Cd, and Pb (mean ± standard deviation, *n* = 3) in samples extracted by microwave-assisted extraction (MAE) using the NADES and AADES (citric acid:xylitol:water (Xyl-CA); citric acid:malic acid:water (MA-CA); and citric acid:β-alanine:water (Ala-CA)).

The solvents prepared by the stirring method showed satisfactory recoveries of 87 and 106% for Cd and Pb, respectively, using the Xyl-CA NADES, 103% for Cd, using the MA-CA NADES, and 100 and 103% for As and Pb, respectively, using the Ala-CA AADES. The solvents prepared by the rotary evaporator method only showed satisfactory recoveries of 86% for As, using Xyl-CA, and 104 and 98% for As and Pb, respectively, using Ala-CA. For the Xyl-CA and MA-CA NADES, the extractive capacity differed according to the preparation method, with the solvents prepared by the stirring method providing better extraction of the analytes. However, for Ala-CA AADES, no difference in the extractive capacity was observed according to the solvent preparation method. Table 7 shows a comparison of the concentration values obtained together with the results for the reference method.

**Table 7:** Comparison of NADES Preparation Methods for the Extraction of As, Cd, and Pb (Mean ± Standard Deviation, *n* = 3) from the Forage Grass Reference Material (B. brizantha cv. Marandu, E1001a)

	concentration (mean ± standard deviation, *n* = 3) (mg kg^–1^)				
method	solvent	As	Cd	Pb	reference
reference value	HNO3	1.69 ± 0.70	19.9 ± 5.1	4.00 ± 1.8	Embrapa
	Xyl-CA	2.03 ± 0.14	21.3 ± 1.3	0.90 ± 0.20	Santana et al. and Guimarães et al.^21,28^
stirring/heating	MA-CA	1.63 ± 0.040	19.1 ± 0.10	4.30 ± 0.20	
	Ala-CA	1.37 ± 0.023			
	Xyl-CA	2.61 ± 0.34	17.35 ± 0.52	4.26 ± 0.37	
stirring	MA-CA	3.04 ± 0.12	20.4 ± 0.53	4.65 ± 0.060	this study
	Ala-CA	1.69 ± 0.050	15.0 ± 0.55	4.13 ± 0.17	
	Xyl-CA	1.45 ± 0.26	9.52 ± 0.68	1.73 ± 0.51	
rotary evaporator	MA-CA	0.47 ± 0.29	4.47 ± 1.1	0.420 ± 0.0020	this study
	Ala-CA	1.76 ± 0.040	14.6 ± 0.59	3.93 ± 0.020	

Comparative evaluation of the results obtained here and those reported by Santana et al. and Guimarães et al.^21,28^ demonstrated the better performance of the solvents prepared in this study for some analytes compared to the solvents prepared by the conventional method of stirring with heating. This was evidenced by the smaller deviations, relative to the reference values, for the concentrations of As using Ala-CA, Cd using MA-CA, and Pb using Xyl-CA, where the solvents were prepared by the stirring method, and for the concentration of As using Xyl-CA prepared by a rotary evaporator. In addition, the findings demonstrated the ability of the Ala-CA solvent to extract Pb from a plant matrix not previously evaluated by Guimarães et al.^28^

Previous studies have investigated the mechanisms responsible for the extraction of metals by solvents formed by different HBDs.^46,47^ It is generally understood that the dissolution of a metal in a DES is governed by proton activity and the complexation capabilities of the DES, which can be modulated by adjusting the types of HBD and HBA and their molar ratios during solvent formation. The nature of the HBD has been shown to strongly influence metal oxide solubility.^48^ Furthermore, it has been demonstrated that higher proton activity in DES composed of weakly complexing HBDs leads to increased metal oxide solubility, due to the capacity of H^+^ to act as an oxygen acceptor.^49^

### 3.3 Ecological Assessment

The energy consumption values for the preparation of the NADES and AADES were 0.0125, 0.0175, and 0.0044 kW h mL^–1^ for the methods using stirring, stirring/heating, and a rotary evaporator under reduced pressure, respectively. All the preparation methods had a low consumption of electricity. In the case of the stirring method, this cost could be reduced further, since the stirrer table had the capacity to simultaneously process up to 25 vials in each batch. Table 8 provides a comparison of the methods, considering energy consumption, maximum production capacity (volume per tube), and analytical frequency.

**Table 8:** Operational Information for the Different NADES and AADES Preparation Methods

method	energy consumption (kW h mL^–1^)	analytical frequency (tube/batch)	maximum production capacity (volume/tube) (mL)
stirring	0.0125	25	250
rotary evaporator	0.0044	1	250
stirring/heating	0.0175	1	400

The EcoScale method is a tool used to evaluate the green/environmentally friendly/sustainable character of chemical processes in a semiquantitative way. This metric gives good scores to processes that prioritize inexpensive compounds, preparation at room temperature, high yield, and safety for the operator and the environment. A score above 75 on the EcoScale represents an excellent green procedure.^27^ The scores obtained for the NADES and AADES prepared by the different methods were 99 (stirring), 97 (stirring/heating), and 98 (rotary evaporator), indicating no significant differences in their green characteristics.

Therefore, the two methods were demonstrated to be able to prepare NADES or AADES by green, sustainable, and low-cost routes. The physicochemical properties (viscosity and melting point) of the solvents showed significant differences among the methods, indicating the possibility of obtaining different characteristics for the same composition and proportion of components by varying the method of preparation. Selection of the method to be used would depend on equipment availability, production scale, preparation time, and analyte (organic or inorganic species) in addition to the desired physicochemical properties.

## 4 Conclusions

Green and sustainable methods were evaluated for the preparation of NADES and AADES, with the ability to modulate the physicochemical properties of the solvents produced. Stirring and rotary evaporator under reduced pressure preparation methods were employed to obtain three different NADESs and AADESs, based on combinations of citric acid, xylitol/malic acid/β-alanine, and water, and they were compared with the method using stirring with heating. A careful characterization of the solvents was made once the formation of the solvents using the stirring and rotary evaporator methods had been confirmed by FTIR and DSC analyses, together with the construction of phase diagrams.

When these solvents were employed in a method for microwave-assisted extraction of a plant material, they showed good capacity to extract Cd and Pb, using NADES prepared by stirring, and As and Pb, using AADES prepared by the stirring and rotary evaporator methods. The stirring and rotary evaporator methods for preparation of NADES and AADES have advantages in terms of efficiency and ease of use when compared to the conventional method of stirring with heating. Elucidation of the mechanisms of interaction between the components for the formation of NADES, the mechanisms of interaction between NADES and elemental analytes, and the factors involved in NADES–sample interactions are steps to be addressed in future studies necessary for the effective implementation of these solvents in sample preparation.

## Supplementary Material







## Notes

Resolution for ideal solubility equation (PDF)
Composition information for the prepared NADES and AADES, instrumental parameters, melting points of the solvents, densities and viscosities of the NADES and AADES, *E*_t_ (30) and λ_max_ values, comparison of the LOD and LOQ values, comparison of NADES preparation methods, and operational information for the different NADES and AADES preparation methods (PDF)
Additional equations (PDF)
