# Supplementary material for: Green Approaches for Preparation of Natural Deep Eutectic Solvents for Determination of As, Cd, and Pb in Plant Samples by ICP-MS
Source: ACS Omega. 2025 Jun 11;10(24):26118–28. doi: 10.1021/acsomega.5c03345 (PMC12199040; doi:10.1021/acsomega.5c03345)
Supplement: Supplementary file 1 [file ao5c03345_si_001.pdf]

Supporting Information

The chemical potential of a component A in an ideal solution at equilibrium is:

$$\mu_A = \mu_A^* + RT \ln ln X_A$$

where μ_A^* is the chemical potential of pure A, R is the gas constant, T the temperature and X_A the mole fraction of A. A solid solute B in contact with A will dissolve until saturation, which is also a state of equilibrium. Therefore, the ideal solubility of B can be estimated through undissolved B ($\mu_B^*(s)$) in equilibrium with the dissolved B in the solution ($\mu_B(l)$).

$$\mu_B^*(s) = \mu_B^*(l) + RT \ln ln X_B \qquad (1)$$

and rearranging

$$\ln ln X_B = \frac{\mu_B^*(s) - \mu_B^*(l)}{RT} =- \frac{\Delta_{fus}G_B}{RT} \qquad (2)$$

where $\Delta_{fus}G_B$ is the Gibbs energy of fusion of pure B. To find the relation between a change of X_B and the resulting change in temperature of solubility, the equation is differentiated at both sides with respect to temperature. Thus, using the Gibbs–Helmholtz equation ($\left(\frac{\partial\left(\Delta_{fus}G_B/T\right)}{\partial T}\right)_P =- \frac{\Delta_{fus}H_B}{T^2}$) we obtain:

$$\frac{d \ln ln X_B}{dT} = \frac{\Delta_{fus}H_B}{RT^2} \qquad (3)$$

Now, integration from $X_B = 1$ (corresponding to $\ln ln X_B = 0$) and T^* being the fusion temperature of pure B gives:

$$\int_0^{\ln ln X_B} d \ln ln X_B = \frac{1}{R}\int_{T^*}^{T} \frac{\Delta_{fus}H_B}{T^2} dT \qquad (4)$$

In many approximations, the enthalpy of fusion is taken as a constant over the range of temperatures involved. On the other hand, $\Delta_{fus}H_B$ can be integrated using the Kirchhoff's law:

$$\Delta_{fus}H_B(T) = \Delta_{fus}H_B\left(T^*\right) + \int_{T^*}^{T} \Delta_{fus}C_{p(B)}dT \qquad (5)$$

but considering that the molar heat capacity of B ($\Delta_{fus}C_{p(B)}$) will vary only slightly (it can be approximated as constant), we have:

$$\Delta_{fus}H_B(T) = \Delta_{fus}H_B\left(T^*\right) + \Delta_{fus}C_{p(B)}\left(T - T^*\right) \qquad (6)$$

and thus, substituting the result of equation (6) in equation (4):

$$\int_{0}^{\ln ln X_B} d\ln ln X_B = \frac{1}{R}\int_{T^*}^{T} \frac{\left[\Delta_{fus}H_B\left(T^*\right)+\Delta_{fus}C_{p(B)}\left(T-T^*\right)\right]}{T^2}dT \qquad (7)$$

$$\ln ln X_B = \frac{1}{R}\left\{\int_{T^*}^{T} \frac{\Delta_{fus}H_B\left(T^*\right)}{T^2}dT + \int_{T^*}^{T} \frac{\Delta_{fus}C_{p(B)}\left(T-T^*\right)}{T^2}dT\right\}$$

$$\ln ln X_B = \frac{1}{R}\left\{\int_{T^*}^{T} \frac{\Delta_{fus}H_B\left(T^*\right)}{T^2}dT + \left[\int_{T^*}^{T} \frac{\Delta_{fus}C_{p(B)}}{T}dT - \int_{T^*}^{T} \frac{\Delta_{fus}C_{p(B)}T^*}{T^2}dT\right]\right\}$$

and finally considering that $\Delta_{fus}C_{p(B)}$ is still a constant:

$$\ln ln X_B = \frac{1}{R}\left\{- \Delta_{fus}H_B\left(T^*\right)\left[\frac{1}{T} - \frac{1}{T^*}\right] + \Delta_{fus}C_{p(B)} \ln ln \frac{T}{T^*} + \Delta_{fus}C_{p(B)}T^*\left[\frac{1}{T} - \frac{1}{T^*}\right]\right\}$$

$$\ln ln X_B = \frac{1}{R}\left\{- \Delta_{fus}H_B\left(T^*\right)\left[\frac{1}{T} - \frac{1}{T^*}\right] + \Delta_{fus}C_{p(B)} \ln ln \frac{T}{T^*} + \Delta_{fus}C_{p(B)}\left[\frac{T^*}{T} - 1\right]\right\}$$

$$\ln ln X_B = -\frac{\Delta_{fus}H_B\left(T^*\right)}{R}\left[\frac{1}{T} - \frac{1}{T^*}\right] + \frac{\Delta_{fus}C_{p(B)}}{R}\ln ln \frac{T}{T^*} + \frac{\Delta_{fus}C_{p(B)}}{R}\left[\frac{T^*}{T} - 1\right]$$

$$\ln ln X_B = -\frac{\Delta_{fus}H_B\left(T^*\right)}{R}\left[\frac{1}{T} - \frac{1}{T^*}\right] + \frac{\Delta_{fus}C_{p(B)}}{R}\left[\ln ln \frac{T}{T^*} + \frac{T^*}{T} - 1\right] \qquad (8)$$
