# Supplementary material for: Green Approaches for Preparation of Natural Deep Eutectic Solvents for Determination of As, Cd, and Pb in Plant Samples by ICP-MS
Source: ACS Omega. 2025 Jun 11;10(24):26118–28. doi: 10.1021/acsomega.5c03345 (PMC12199040; doi:10.1021/acsomega.5c03345)
Supplement: Supplementary file 2 [file ao5c03345_si_002.pdf]

Table 1. Composition information for the prepared NADES and AADES.

| Solvent | Mass ratio (% w $w^{-1}$) | Molar ratio | Composition |
|---|---|---|---|
| Xyl-CA NADES | 42:13:45 | 3:1:30 | Citric acid: xylitol: water |
| MA-CA NADES | 42:13:45 | 2:1:26 | Citric acid: malic acid: water |
| Ala-CA AADES | 43.75:12.5:43.75 | 2:1:17 | Citric acid: β-alanine: water |

Table 2. Instrumental parameters for the analysis of $^{75}As^{+}$, $^{111}Cd^{+}$, and $^{208}Pb^{+}$ by ICP-MS.

| **Instrumental parameters** | |
|---|---|
| Radiofrequency power | 1600 W |
| Plasma gas flow | 18 L $min^{-1}$ |
| Auxiliary gas flow | 1.2 L $min^{-1}$ |
| Nebulizer gas flow | 1.0 mL $min^{-1}$ |
| Sample uptake rate | 0.70 mL $min^{-1}$ |
| **Method parameters** | |
| Sweeps/reading | 50 |
| Readings/replicate | 1 |
| Replicates | 3 |
| Dwell time | 25 s |
| RPq [a] | 0.25 |
| Analytical calibration range | 0.50 – 20 µg $L^{-1}$ |

[a] RPq: quadrupole dynamic bandpass tuning parameter.

Table 3. Melting points of the solvents prepared by the three methods.

| | Melting point (K) | | |
|---|---|---|---|
| **Solvent** | Stirring | Stirring/heating | Rotary evaporator |
| Xyl-CA NADES | 245 | 242 | - |
| MA-CA NADES | 240 | 238 | - |
| Ala-CA AADES | 251 | 251 | 223 |

Table 4. Densities and viscosities of the NADES and AADES prepared at a temperature of 24 ºC (mean ± standard deviation, n = 3).

| | **Xyl-CA NADES** | |
|---|---|---|
| **Preparation method** | **Density (g mL$^{-1}$)** | **Viscosity (mPa s)** |
| Stirring | 1.25 ± 0.01 | 7.46 ± 0.02 |
| Stirring/heating | 1.26 ± 0.01 | 6.90 ± 0.01 |
| Rotary evaporator | 1.30 ± 0.01 | 21.5 ± 0.05 |
| | **MA-CA NADES** | |
| **Preparation method** | **Density (g mL$^{-1}$)** | **Viscosity (mPa s)** |
| Stirring | 1.26 ± 0.01 | 5.99 ± 0.02 |
| Stirring/heating | 1.27 ± 0.01 | 5.83 ± 0.05 |
| Rotary evaporator | 1.34 ± 0.01 | 18.9 ± 0.3 |
| | **Ala-CA AADES** | |
| **Preparation method** | **Density (g mL$^{-1}$)** | **Viscosity (mPa s)** |
| Stirring | 1.26 ± 0.01 | 10.9 ± 0.01 |
| Stirring/heating | 1.27 ± 0.01 | 11.2 ± 0.08 |
| Rotary evaporator | 1.30 ± 0.01 | 26.2 ± 0.2 |

Table 5. Et (30) and $\lambda_{max}$ values for the NADES and AADES prepared by the different methods.

| | Stirring | | Stirring/heating | | Rotary evaporator | |
|---|---|---|---|---|---|---|
| Solvent | λ (nm) | Et (30) (kcal mol$^{-1}$) | λ (nm) | Et (30) (kcal mol$^{-1}$) | λ (nm) | Et (30) (kcal mol$^{-1}$) |
| Xyl-CA NADES | 306 | 93.6 | 305 | 93.7 | 303 | 94.3 |
| MA-CA NADES | 303 | 94.5 | 302 | 94.7 | 300 | 95.5 |
| Ala-CA AADES | 304 | 94.2 | 304 | 94.0 | 304 | 94.2 |

Table 6. Comparison of the LOD and LOQ values for As, Cd and Pb determination by ICP-MS after extraction using microwave-assisted acid digestion (MW-AD) and microwave-assisted extraction (MAE) with NADES and AADES prepared by stirring, stirring/heating and rotary evaporator.

| | | | As | | Cd | | Pb | | |
|---|---|---|---|---|---|---|---|---|---|
| Solvent Preparation Method | Solvent | Sample Preparation Method | LOD (mg $kg^{-1}$) | LOQ (mg $kg^{-1}$) | LOD (mg $kg^{-1}$) | LOQ (mg $kg^{-1}$) | LOD (mg $kg^{-1}$) | LOQ (mg $kg^{-1}$) | Reference |
| -- | $HNO_3$ | MW-AD | 0.0001 | 0.0006 | 0.0009 | 0,003 | 0.08 | 0.2 | Brito (2025) |
| Stirring/heating | Xyl-CA | MAE | 0.008* | 0.03* | 0.05* | 0,2* | 1.3* | 4.3* | Santana (2020) and Guimarães (2023) |
| | MA-CA | | 0.02* | 0.07* | 0.002* | 0.006* | 2.6* | 7.1* | |
| | Ala-CA | | 0.02 | 0.05 | -- | -- | -- | -- | |
| Stirring | Xyl-CA | MAE | 0.01 | 0.04 | 0.01 | 0.02 | 0.01 | 0.4 | This study |
| | MA-CA | | 0.06 | 0.2 | 0.01 | 0.02 | 0.08 | 0.2 | |
| | Ala-CA | | 0.02 | 0.06 | 0.02 | 0.05 | 0.01 | 0.04 | |
| Rotary evaporator | Xyl-CA | MAE | 0.1 | 0.4 | 0,02 | 0.05 | 0.01 | 0.04 | This study |
| | MA-CA | | 0.08 | 0.2 | 0.01 | 0.02 | 0.04 | 0.1 | |
| | Ala-CA | | 0.01 | 0.03 | 0.01 | 0.02 | 0.07 | 0.02 | |

Data: * Values in µg $kg^{-1}$.

Table 7. Comparison of NADES preparation methods for the extraction of As, Cd, and Pb (mean ± standard deviation, n = 3) from the forage grass reference material (*Brachiaria brizantha* cv. Marandu, Embrapa E1001a).

| | | Concentration (mean ± standard deviation, n = 3) (mg kg$^{-1}$) | | | |
|---|---|---|---|---|---|
| Method | Solvent | As | Cd | Pb | Reference |
| Reference value | $HNO_3$ | 1.69 ± 0.70 | 19.9 ± 5.1 | 4.0 ± 1.8 | Embrapa |
| Stirring/heating | Xyl-CA | 2.03 ± 0.14 | 21.3 ± 1.3 | 0.9 ± 0.20 | Santana et al. and Guimarães et al. [21,28] |
| | MA-CA | 1.63 ± 0.040 | 19.1 ± 0.10 | 4.3 ± 0.20 | |
| | Ala-CA | 1.37 ± 0.023 | ----- | ----- | |
| Stirring | Xyl-CA | 2.61 ± 0.34 | 17.35 ± 0.52 | 4.26 ± 0.37 | This study |
| | MA-CA | 3.04 ± 0.12 | 20.4 ± 0.53 | 4.65 ± 0.060 | |
| | Ala-CA | 1.69 ± 0.050 | 15.0 ± 0.55 | 4.13 ± 0.17 | |
| Rotary evaporator | Xyl-CA | 1.45 ± 0.26 | 9.52 ± 0.68 | 1.73 ± 0.51 | This study |
| | MA-CA | 0.47 ± 0.29 | 4.47 ± 1.1 | 0.420 ± 0.0002 | |
| | Ala-CA | 1.76 ± 0.040 | 14.6 ± 0.59 | 3.93 ± 0.020 | |

Table 8. Operational information for the different NADES and AADES preparation methods.

| Method | Energy consumption (kWh mL$^{-1}$) | Analytical frequency (tube/batch) | Maximum production capacity (volume/tube) (mL) |
|---|---|---|---|
| Stirring | 0.0125 | 25 | 250 |
| Rotary evaporator | 0.0044 | 1 | 250 |
| Stirring/heating | 0.0175 | 1 | 400 |
