# Supplementary material for: Green Approaches for Preparation of Natural Deep Eutectic Solvents for Determination of As, Cd, and Pb in Plant Samples by ICP-MS
Source: ACS Omega. 2025 Jun 11;10(24):26118–28. doi: 10.1021/acsomega.5c03345 (PMC12199040; doi:10.1021/acsomega.5c03345)
Supplement: Supplementary file 3 [file ao5c03345_si_003.pdf]

Green approaches for preparation of Natural Deep Eutectic Solvents for determination of As, Cd, and Pb in plant samples by ICP-MS

Sabrina S. Ferreira[a], Rafaela S. Lamarca[a], Leandro S. Silva[a], Thiago A. L. Burgo[c], Paulo C. F. Lima Gomes[d], Clarice D. B. Amaral[e], Jose O. Fernandes[f], Sara C. Cunha[f], Mario H. Gonzalez[a*]

[a] São Paulo State University (UNESP), Department of Chemistry and Environmental Science, National Institute for Alternative Technologies of Detection, Toxicological Evaluation and Removal of Micropollutants and Radioactives (INCT-DATREM), São José do Rio Preto, SP, 15054-000, Brazil

[c] Department of Chemistry and Environmental Science, São Paulo State University (UNESP), São José do Rio Preto, SP, 15054-000, Brazil

[d] Department of Analytical Chemistry, Physical Chemistry and Inorganic Chemistry, National Institute for Alternative Technologies of Detection, Toxicological Evaluation and Removal of Micropollutants and Radioactives (INCT-DATREM), Institute of Chemistry, São Paulo State University (UNESP), Araraquara, SP, 14800-060, Brazil

[e] Department of Chemistry, Federal University of Paraná (UFPR), Curitiba, PR, 81531-980, Brazil

[f] LAQV-REQUIMTE, Laboratory of Bromatology and Hydrology, Faculty of Pharmacy, University of Porto, Portugal

* Corresponding author

E-mail address: mario.gonzalez@unesp.br (M.H. Gonzalez)

Supporting Information

The chemical potential of a component *A* in an ideal solution at equilibrium is:

$$\mu_A = \mu_A^* + RT\,ln\,X_A$$

where μ_A^* is the chemical potential of pure *A*, *R* is the gas constant, *T* the temperature and X_A the mole fraction of *A*. A solid solute *B* in contact with *A* will dissolve until saturation, which is also a state of equilibrium. Therefore, the ideal solubility of *B* can be estimated through undissolved *B* ($\mu_B^*(s)$) in equilibrium with the dissolved *B* in the solution ($\mu_B(l)$).

$$\mu_B^*(s) = \mu_B^*(l) + RT\,ln\,X_B \tag{1}$$

and rearranging

$$ln\,X_B = \frac{\mu_B^*(s) - \mu_B^*(l)}{RT} = -\frac{\Delta_{fus}G_B}{RT} \tag{2}$$

where $\Delta_{fus}G_B$ is the Gibbs energy of fusion of pure *B*. To find the relation between a change of X_B and the resulting change in temperature of solubility, the equation is differentiated at both sides with respect to temperature. Thus, using the Gibbs–Helmholtz equation ($\left(\frac{\partial\left(\Delta_{fus}G_B/T\right)}{\partial T}\right)_P = -\frac{\Delta_{fus}H_B}{T^2}$) we obtain:

$$\frac{d\,ln\,X_B}{dT} = \frac{\Delta_{fus}H_B}{RT^2} \tag{3}$$

Now, integration from $X_B = 1$ (corresponding to $ln\,X_B = 0$) and T^* being the fusion temperature of pure *B* gives:

$$\int_0^{ln\,X_B} d\,ln\,X_B = \frac{1}{R}\int_{T^*}^{T} \frac{\Delta_{fus}H_B}{T^2}dT \tag{4}$$

In many approximations, the enthalpy of fusion is taken as a constant over the range of temperatures involved. On the other hand, $\Delta_{fus}H_B$ can be integrated using the Kirchhoff's law:

$$\Delta_{fus}H_B(T) = \Delta_{fus}H_B\left(T^*\right) + \int_{T^*}^{T} \Delta_{fus}C_{p(B)}dT \qquad (5)$$

but considering that the molar heat capacity of B ($\Delta_{fus}C_{p(B)}$) will vary only slightly (it can be approximated as constant), we have:

$$\Delta_{fus}H_B(T) = \Delta_{fus}H_B\left(T^*\right) + \Delta_{fus}C_{p(B)}\left(T - T^*\right) \qquad (6)$$

and thus, substituting the result of equation (6) in equation (4):

$$\int_{0}^{ln\,X_B} d\,ln\,X_B = \frac{1}{R}\int_{T^*}^{T} \frac{\left[\Delta_{fus}H_B\left(T^*\right)+\Delta_{fus}C_{p(B)}\left(T-T^*\right)\right]}{T^2}dT \qquad (7)$$

$$ln\,X_B = \frac{1}{R}\left\{\int_{T^*}^{T} \frac{\Delta_{fus}H_B\left(T^*\right)}{T^2}dT + \int_{T^*}^{T} \frac{\Delta_{fus}C_{p(B)}\left(T-T^*\right)}{T^2}dT\right\}$$

$$ln\,X_B = \frac{1}{R}\left\{\int_{T^*}^{T} \frac{\Delta_{fus}H_B\left(T^*\right)}{T^2}dT + \left[\int_{T^*}^{T} \frac{\Delta_{fus}C_{p(B)}}{T}dT - \int_{T^*}^{T} \frac{\Delta_{fus}C_{p(B)}T^*}{T^2}dT\right]\right\}$$

and finally considering that $\Delta_{fus}C_{p(B)}$ is still a constant:

$$ln\,X_B = \frac{1}{R}\left\{-\Delta_{fus}H_B\left(T^*\right)\left[\frac{1}{T} - \frac{1}{T^*}\right] + \Delta_{fus}C_{p(B)}\,ln\,\frac{T}{T^*} + \Delta_{fus}C_{p(B)}T^*\left[\frac{1}{T} - \frac{1}{T^*}\right]\right\}$$

$$ln\,X_B = \frac{1}{R}\left\{-\Delta_{fus}H_B\left(T^*\right)\left[\frac{1}{T} - \frac{1}{T^*}\right] + \Delta_{fus}C_{p(B)}\,ln\,\frac{T}{T^*} + \Delta_{fus}C_{p(B)}\left[\frac{T^*}{T} - 1\right]\right\}$$

$$ln\,X_B = -\frac{\Delta_{fus}H_B\left(T^*\right)}{R}\left[\frac{1}{T} - \frac{1}{T^*}\right] + \frac{\Delta_{fus}C_{p(B)}}{R}\,ln\,\frac{T}{T^*} + \frac{\Delta_{fus}C_{p(B)}}{R}\left[\frac{T^*}{T} - 1\right]$$

$$ln\,X_B = -\frac{\Delta_{fus}H_B\left(T^*\right)}{R}\left[\frac{1}{T} - \frac{1}{T^*}\right] + \frac{\Delta_{fus}C_{p(B)}}{R}\left[ln\,\frac{T}{T^*} + \frac{T^*}{T} - 1\right] \qquad (8)$$
